# Treatment Efficiency of Maxillary and Mandibular Orovestibular Tooth Expansion and Compression Movements with the Invisalign^®^ System in Adolescents and Adults

**DOI:** 10.3390/jcm13051267

**Published:** 2024-02-23

**Authors:** Ludger Keilig, Lena Brieskorn, Jörg Schwarze, Werner Schupp, Christoph Bourauel, Anna Konermann

**Affiliations:** 1 Oral Technology, University Hospital Bonn, 53111 Bonn, Germany; 2 Department of Prosthodontics, University Hospital Bonn, 53127 Bonn, Germany; 3 Private Practice, 50674 Cologne, Germany; 4 Private Practice, 50996 Cologne, Germany; 5 Department of Orthodontics, University Hospital Bonn, University of Bonn, Welschnonnenstr. 17, 53111 Bonn, Germany

**Keywords:** aligner, Invisalign^®^, orovestibular tooth movement, orthodontics, treatment efficiency

## Abstract

Objectives: Aligners are an effective and esthetic orthodontic treatment option for permanent and mixed dentition. There are only a few studies dealing with the effectiveness of orovestibular tooth movement using aligners and applying adequate examination methods. In the present retrospective study, the aligner efficiency of orovestibular movements for the entire dentition was systematically evaluated using 3D superimposition, taking into account the influence of jaw, tooth type and Invisalign^®^ system. Methods: Group 1 (*n* = 18 adults, Invisalign^®^) and Group 2 (*n* = 17 adolescents, Invisalign^®^ Teen) were treated with Invisalign^®^ Ex30 aligner material and Invisalign^®^ specific auxiliary means. In this non-interventional retrospective study, pre- and post-treatment maxillary and mandibular plaster cast models were scanned and superimposed with ClinChecks^®^ via Surface–Surface Matching Algorithm on unmoved teeth providing stable references. Effectivity of planned versus clinically realized movements was evaluated for each tooth. Statistics were performed with a *t*-test and Bonferroni–Holm correction (*α* = 0.05). Results: Orovestibular movement efficiency was excellent without statistical significance regarding jaw, tooth type or Invisalign^®^ system. Mandibular translational tooth movements were highly effective, and outstanding for premolars (91–98%). Maxillary translational tooth movements were successful for incisors and premolars, but less effective for canines and molars. Almost all teeth were moderately or very effectively corrected by crown tipping, performing better for mandibular (70–92%) than maxillary (22–31%) canines as much as for adolescent upper front teeth (81–85%) and lower canines (92%). Conclusions: Aligners are able to effectively implement translational orovestibular movements, supported by tilting the crowns for even more efficient implementation of the movements. This phenomenon was observed in our studies for all teeth in both jaws, regardless of the Invisalign^®^ system used. Treatment planning should nevertheless take into account the individual patient parameters with regard to the movements to be performed in order to make the aligner therapy as successful as possible in terms of realizing the desired therapeutic goal.

## 1. Introduction

Clear aligners have emerged as validated orthodontic treatment modality worldwide in the past two decades and still gain increasing popularity, particularly owing to their esthetic benefits. Generally, each aligner can affect tooth movements of 0.25 to 0.3 mm and of 2° on average [1]. In order to achieve these movements, aligner therapy requires higher patient compliance compared to multibracket therapy due to the removability of the devices, but the advantages outweigh the disadvantages, particularly from a patient perspective [2]. To mention the most relevant aspects, dental hygiene, caries control and speaking without limitations are possible and devoid of difficulty, and furthermore the high wearing comfort as much as the invisibility of the appliances make aligners attractive not only for adults but also for adolescents [3,4,5,6,7,8]. Since 2009, the option of aligner treatment is no longer restricted to adults or adolescents with full second dentition, but also available for teenagers with mixed dentition and erupting teeth due to the launch of Invisalign^®^ Teen by Align Technology Inc. (Santa Clara, CA, USA). Investigations revealed that teenagers treated with aligners feature better compliance in oral hygiene, less plaque, and fewer gingival inflammatory reactions than the ones under fixed appliance therapy [3]. However, no data on the effectiveness of treatment with Invisalign^®^ Teen exist to date, as such studies only focused on the compliance of adolescents wearing aligners [8,9].

Initially, aligners were considered a therapy device for correction of minor malpositioned teeth, but not for complex cases [10]. To date, the range of indications has been successively expanded through continuous advancements of the system [11,12,13]. In the current literature, clear aligner treatment is already estimated equivalent to the multi-bracket appliance as the gold standard in mild and moderate cases, but it is nonetheless an ongoing discussion whether aligner treatment is capable of perfectly correcting complex malocclusions as well [10,14,15]. A major point for skepticism is the assumption that the system most likely induces a tipping of moved teeth rather than a bodily translation, which represents a disadvantage compared to brackets [16,17]. In order to overcome this burden, additional tools have been developed for supporting the intended tooth movements, namely composite attachments on buccal and lingual tooth surfaces and power ridge points imprinted in the aligners that modify force projection [18,19,20]. When chronologically reflecting the weak data availability in the literature on aligner treatment spectrum and effectiveness, it was stated in 2000 that aligners enable correction of mild to moderate interproximal spaces and crowding, and that experts might be capable of conducting dental expansions, class II/III corrections and space closure upon extraction [21]. In the following years, investigations on the realization of specific tooth movements such as intrusion, rotation, incisor torque or molar distalization have been conducted, many of them implementing the impact of attachments and power ridges on the overall accuracy of treatment outcomes [18,19,22]. Just recently, a few studies started to focus on the feasibility and predictability of arch expansion with aligners [5,23,24,25]. However, these works manifested limitations regarding the methodologies applied and the overall study design. Mostly, linear measurements on digital arch models were performed, which is considerably defective due to the disregard of three-dimensionality. Other works applied the gingival margin as reference topology even though this structure is virtually designed in the ClinCheck^®^ or did not directly correlate 3D models and ClinChecks^®^.

In order to overcome these burdens, superimposition techniques have to be used, which were implemented for the first time in aligner research by Kravitz et al. in 2009 [26]. By superimposing predicted tooth movements of the ClinCheck^®^ onto achieved tooth positions of virtual models with Invisalign^®^’s proprietary superimposition software ToothMeasure, potential discrepancies could be analyzed with an accuracy of 0.2 mm and 1.0° [26]. However, the major limitation of this study was the fact that only cases with corrections in the front region were incorporated and posterior tooth movements were disregarded owing to the necessity to superimpose the models on stationary teeth.

Our investigation is the first work that focuses on bridging this gap, combining both application of superimposition techniques and investigation on the whole jaws with anterior and posterior maxillary and mandibular regions. In the present retrospective study, we were able to combine both aspects with an advanced and seminal superimposition technique. The first aim of our investigation was to evaluate aligner efficiency of arch expanding and compressing tooth movements for all teeth of upper and lower jaws with this methodology. Secondly, we focused on analyzing potential differences in aligner performance with regard to upper and lower jaw and to tooth specimen for these orovestibular movements. Finally, we pursued the question whether treatment results are impacted by the aligner version, namely the conventional Invisalign^®^ system versus Invisalign^®^ Teen. In summary, the basic idea of our study is to evaluate the effects of the three aspects investigated, namely jaw, tooth group and aligner system, on the efficiency of orovestibular tooth movement. Our null hypothesis is that there are no statistically significant differences between these aspects. Our alternative hypothesis is that there may be statistically significant differences in treatment outcomes based on jaw, tooth group and aligner system.

## 2. Materials and Methods

### 2.1. Patient Collective

The data and clinical models were obtained from a patient collective, randomly selected without specification of the initial malocclusion or the amount of planned tooth correction in order to receive a broad spectrum of cases, subdivided into 2 groups. The study was conducted in accordance with the Declaration of Helsinki. The protocol did not need approval by the Ethics Committee of the University of Bonn since this was a non-interventional retrospective study. The patients had already finalized their treatment according to their orthodontic indication before the beginning of this study and had given their written consent to the treatment in advance. 

Group 1 incorporated 18 adult patients treated with the conventional Invisalign^®^ system, and Group 2 comprised 17 adolescent participants treated with Invisalign^®^ Teen. A complete analysis for evaluation of the necessary number of patients was not possible, as for these concrete clinical questions no previous statistical data exist in the literature. Patient selection criteria included good general health, no medication affecting bone or soft tissue metabolism, no prosthetic restorations on the moved teeth, no premature occlusal contact on the front teeth, no radiographic signs of horizontal bone loss or vertical bony defects and no manifestations of root resorptions. All patients were treated with Invisalign^®^ Exceed30 (EX30) aligner material and received Invisalign^®^ specific auxiliary means such as attachments according to their individual needs. The number of aligners was prescribed by the malocclusion and varied between individuals.

### 2.2. Data Selection and Preparation

**Figure 1:**
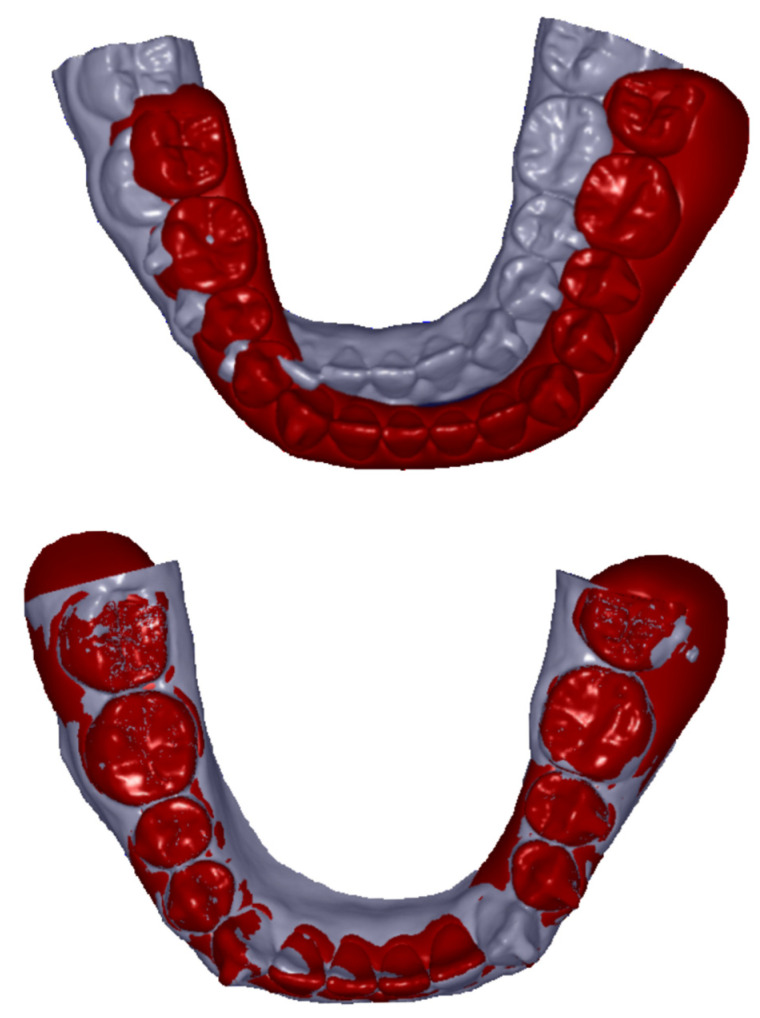
Representative example of a ClinCheck^®^ (red, presented as surface) and a corresponding plaster cast model (grey, presented as surface) of the initial situation before treatment from a lower jaw. The upper picture shows the condition before matching and the lower picture represents the same situation after matching on the unmoved lower first and second premolars and the first molars in both quadrants.

Data and documents for analyses comprised plaster cast models of upper and lower jaws from the initial (M.in) and the final situation after end of treatment (M.fin) for each patient. Here, only cases with at least 3 unmoved, not neighboring teeth per jaw were included in order to guarantee a stable superimposition. Plaster casts were digitized with a laser scanner (Micromeasure^®^ 70, Micromeasure GmbH, Bischoffen, Germany). Models were fixed on an adjustable, motorized stage guided via the software ‘ScanOs’(micromeasure GmbH), enabling digitization of even undercut areas. To achieve a perfect digitization, each model was scanned from four different perspectives. Resulting scatterplots were reduced to areas with teeth and gingiva and subsequently overlayed to a common surface. Achieved data were exported to an ASCII text data file. According to the manufacturer, distances between laser lines measure 100 µm on the x-axis and 53 µm on the y-axis. The data of the virtually planned treatment goals, the digital ClinChecks^®^ (C), were provided as STL files by Align Technology Inc., consolidated for upper and lower jaws. The program ReMESH (version 2.1, Marco Attene, Istituto di Matematica Applicata e Tecnologie Informatiche Consiglio Nazionale delle Ricerche, Genova, Italy) was used to unravel the data for both jaws separately and to save them as independent files. Superimpositions of the three aforementioned digital data sets (M.in, M.fin, C) were realized by overlaying the models on the teeth that were not moved and thus served as stable references (Figure 1). After application of this selection criterion, 7 upper and 4 lower jaw models of Group 1 and 5 upper and 4 lower jaw models of Group 2 had to be excluded, as in these cases all teeth had to be moved according to the treatment plan. In addition, some models had to be eliminated due to inaccuracies or defects in the material. Thus, the overall collective used for analyses comprised 10 upper and 10 lower jaw models for Group 1 and 12 upper and 11 lower jaw models for Group 2.

### 2.3. Methodology of Investigations

Digitized models and ClinChecks^®^ were visualized and edited with a specific 3D graphic software (Surfacer 10.5, Imageware/Siemens PLM Software, Plano, TX, USA), segmenting each tooth from the scatterplot surfaces before replicating it. Finally, complete model surfaces with alveolar ridges and separate tooth surfaces were available for each tooth. All teeth were investigated except the second and third molars. Superimpositions were realized with the Surface–Surface Matching Algorithm [27], overlaying the unmoved teeth of the final situation and the ClinCheck^®^, respectively, according to the principle of distance minimizing on the unmoved teeth of the initial situation (Figure 1). This results in a common reference system to which the rest of the alveolar ridge can be matched. It is generally known that even unmoved teeth can change their initial position and slightly move during orthodontic treatment due to interarch forces. Therefore, we verified whether the intertooth distance from the unmoved teeth differed from those of the final ClinCheck^®^, and excluded those unmoved teeth without a perfect fit in both categories.

After the general superimposition of the corresponding digital models in the different stages, we used the following procedure to determine the origin for the local coordinate system of each crown separately. First, we segmented the corresponding crown from the M.in model. Then we determined the minimum and maximum x and y coordinates over all points within the point set of this crown, and used x_0_ = (x_max_ + x_min_)/2 and y_0_ = (y_max_ + y_min_)/2 as the x and y coordinates for the local crown origin. This is basically the center of a 2D bounding box parallel to the occlusal plane. Finally, we determined the maximum z coordinate over all points within the point set of this crown, and used z_0_ = z_max_ as the z coordinate for the local crown origin.

Consequently, for analyses of single tooth movements, we specified a point in the center of the occlusal plane for each single tooth crown from the initial model M.in used as local coordinate origin. This local origin was then used in the subsequent superimposition of the corresponding crowns in the different models. A superimposition of individual scatterplots was conducted to calculate the clinically realized tooth movements by adjustment from M.in to C. The resulting differences in tooth positions calculated via Excel 2010 (Microsoft Corporation, Remond, WA, USA) gave information on six types of movement of the crowns: translations along the x-, y- and z-axis (Tx, Ty, Tz) and rotations around the x-, y- and z-axis (Rx, Ry, Rz). As movements of the roots were not retraceable with the given information, no differences were made between controlled and uncontrolled tipping and torque. 

**Figure 2:**
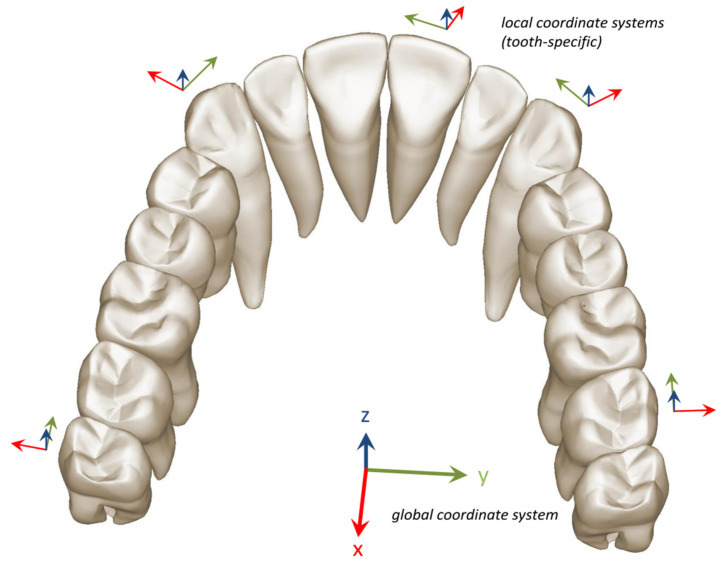
Illustration of an exemplary upper arch with global coordinate system (tall) and several tooth-specific coordinate systems (small). Tooth movements with course on the tooth-specific coordinate system along the x-axis correspond to mesio-distal movements, along the y-axis to orovestibular movements and along the z-axis to intrusions versus extrusions.

In order to equalize measurement results, a right-handed cartesian coordinate system with orthogonal coordinate axes was assigned to each model by which they were aligned in the Surfacer software (Surfacer 10.5, Imageware/Siemens PLM Software, Plano, TX, USA). Instead of the longitudinal axis of the tooth, which is not determinable via ClinCheck^®^, we used the global z-axis from this coordinate system established by means of a plane equaling the occlusal plane. As tooth-specific movements should be evaluated, the right-handed model-specific coordinate system had to be transformed into a tooth-specific coordinate system (Figure 2). For this, the x-axis was assigned to the transversal, the y-axis to the sagittal and the z-axis to the vertical direction.

### 2.4. Data Analysis and Statistics

Effectivity of planned compared to clinically realized movements was evaluated for each tooth. Only teeth that showed planned translations above 0.2 mm and/or planned rotations above 2° were included in the following analysis. This was done as inaccuracies in the digitalization process in combination with inaccuracies of the proposed method result in high relative differences for small tooth movements. Including such small tooth movements would negatively influence the signal-to-noise ratio in the gathered data. Analyses were conducted if the sample size incorporated 3 cases or more. Tooth movements were separately calculated for each tooth from first incisor to the first molar of the upper and lower jaw, where first and second quadrant and third and fourth quadrant were taken together. In addition, we did not differentiate between expansion and compression-effecting movements and regarded them together as orovestibular movements.

The accuracy of the determined tooth movements depends on the extent of the movement. Systematic errors, either from the digitalization process or from methodical errors, influence small movements more than large movements. This holds especially when determining the efficiency as the quotient of the realized movements divided by the planned movements, i.e.,

$$e=\frac{m^{real}}{m^{plan}}$$

To compensate for this, we used the method of weighted means. Basically, this assumes that each calculated efficiency itself can be considered as a mean value over a single value, each with a separately assigned weight representing the reliability of these data. For each efficiency we used the general weighted means formula

$$\overset{¯}{e}=\frac{\sum_{i=0}^{n}w_{i}\cdot e_{i}}{\sum_{i=0}^{n}w_{i}}$$

to determine the mean values $\overset{¯}{e}$, and chose the weights as $w_{i}=\left|m_{i}^{plan}\right|$. 

Statistics were performed for three different aspects:

1. Whether there were significant differences in the realization of expansion and compression movements with regard to the tooth type, namely incisors, canines, premolars and molars.
2. Whether the fact that bone structure differs in maxilla and mandibula was reflected by different effectivity of these orovestibular movements.
3. Whether due to either aspect 1 or 2, Group 1 and Group 2 exhibit significant variances.

**Figure 3:**
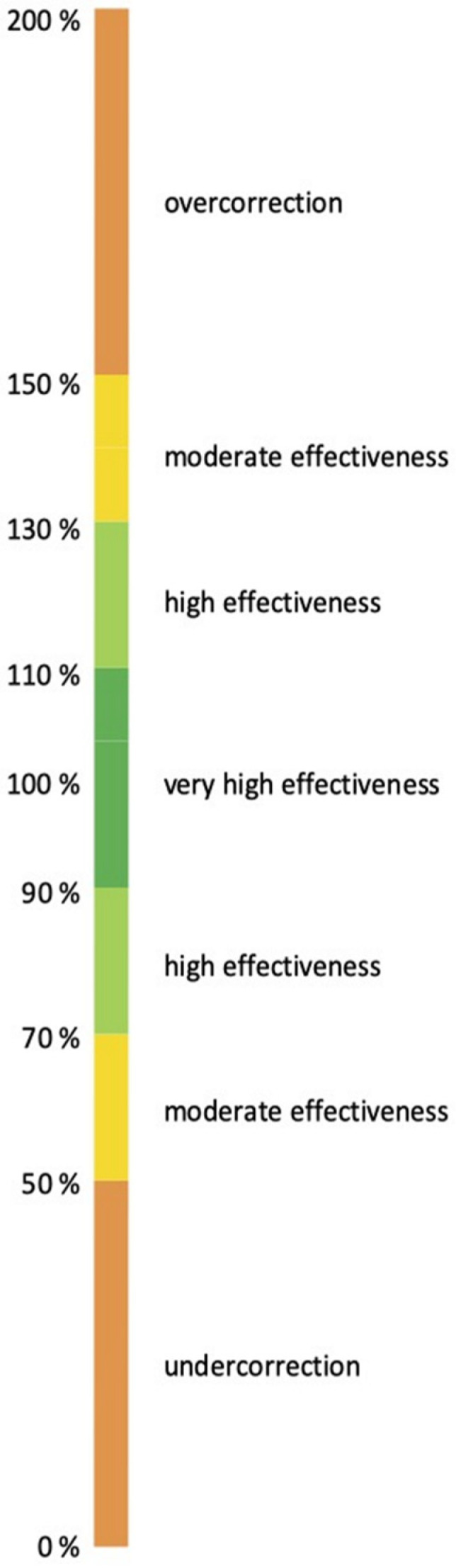
Rating scale for the categorization of results. Colored bars correlate with the mean relative effectivity and its corresponding interpretations. Values between 0 and 49% indicate a correction below the expected result, 50–69% and 131–150% a moderate effectivity, 70–89% and 111–130% a high effectivity, 91–110% a very high effectivity and 151% or higher an overcorrection.

Statistical analyses were done with a *t*-test and subsequent correction according to Bonferroni–Holm. A significance level of α = 0.05 was considered statistically significant. A rating scale visualized in Figure 3 was used to categorize results to simplify and standardize an objective nomenclature. Values between 0 and 49% indicate a correction below the expected result, 50–69% and 131–150% a moderate effectivity, 70–89% and 111–130% a high effectivity, 91–110% a very high effectivity and 151% or higher an overcorrection.

## 3. Results

### 3.1. Effectiveness of Translational Movements for the Different Tooth Types of Upper and Lower Jaws

**Figure 4:**
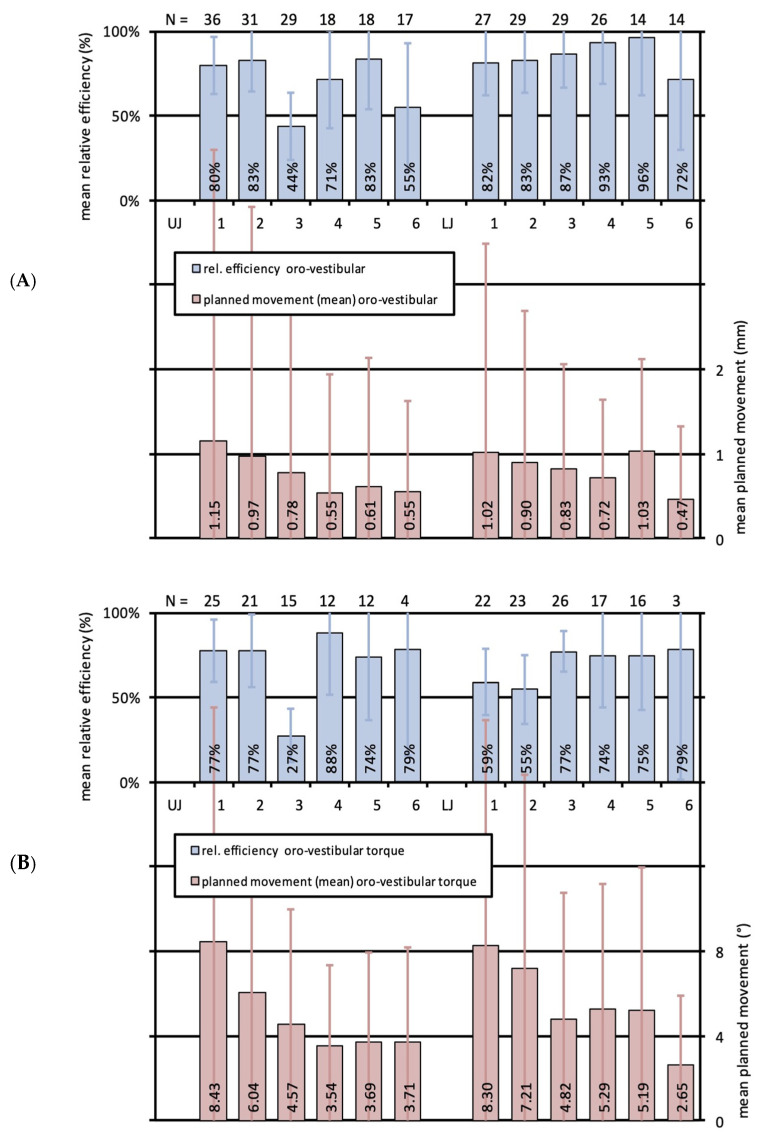
(**A**) Bar graph illustrating the relative effectivity of translational orovestibular tooth movements for the whole study cohort. Upper bars (blue) indicate the relative effectivity and the error in percent for each tooth of the upper (UJ, left) and the lower (LJ, right) jaw. Lower bars (red) show the mean planned movements in mm with standard deviations (SD). N-numbers of the corresponding mean values are specified on top of the illustration. (**B**) Bar graph illustrating the relative effectivity of rotations around the y-axis, equivalent to orovestibular torque, for the whole study cohort. Upper bars (blue) indicate the relative effectivity and the error in percent for each tooth of the upper (UJ, left) and the lower (LJ, right) jaw. Lower bars (red) show the mean planned movements in mm with standard deviations (SD). N-numbers of the corresponding mean values are specified on top of the illustration.

Most extent orovestibular movements were planned for upper and lower first incisors with 1.0 mm and 1.2 mm, and featured efficiencies of 80% and 82%, respectively (Figure 4A). Lower second premolars showed a very high effectivity of 96% for a similar amount of movement, and lower first premolars also presented a very high effectivity of 93% for 0.7 mm planned movement. Second incisors of both jaws, upper premolars, lower canines and lower molars manifested a high effectivity between 71 and 87% for mean planned movements of 0.5–1.0 mm. Exclusively the upper canines with 44% and upper molars with 55% could only reach a correction below the expected result or a moderate effectivity for 0.8 and 0.5 mm movement, respectively. Statistical outcomes did not significantly differ among single teeth or between upper and lower jaws.

### 3.2. Effectiveness of Orovestibular Tipping for the Different Tooth Types of Upper and Lower Jaws

Planned orovestibular crown tipping resulted in large standard deviations for all teeth investigated except for the first molars, which is illustrated in Figure 4B. Most extent movements were again seen for upper and lower first incisors with 8.4° and 8.3°. Regarding first and second incisors together, upper front teeth seemed to superiorly implement tipping with an effectivity of 77% compared to the lower ones with 59% and 55%. A high effectivity in orovestibular crown tipping was seen for upper and lower premolars and molars and for lower canines with 74–88%. Contrarily, upper canines were corrected below the expected result with 27% for planned movements of 4.6°. Statistical analyses revealed no significant differences among single teeth or between upper and lower jaws.

### 3.3. Effectiveness of Translational Movements in Upper and Lower Jaws for Adolescent and Adult Patients

**Figure 5:**
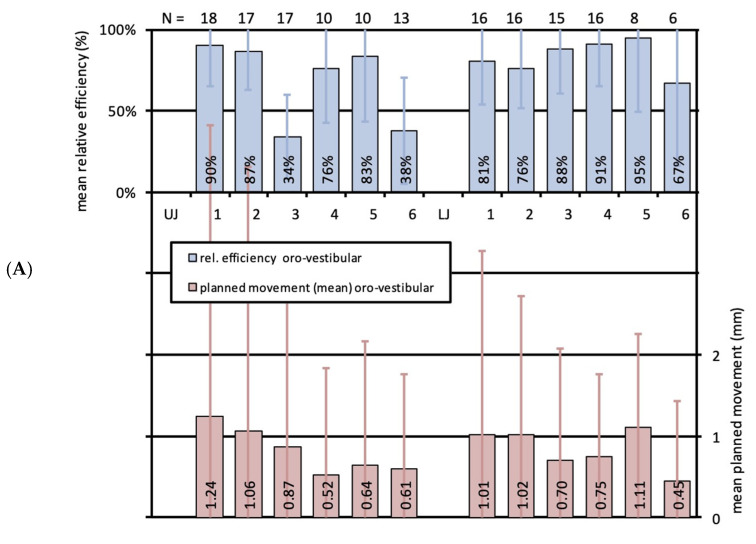

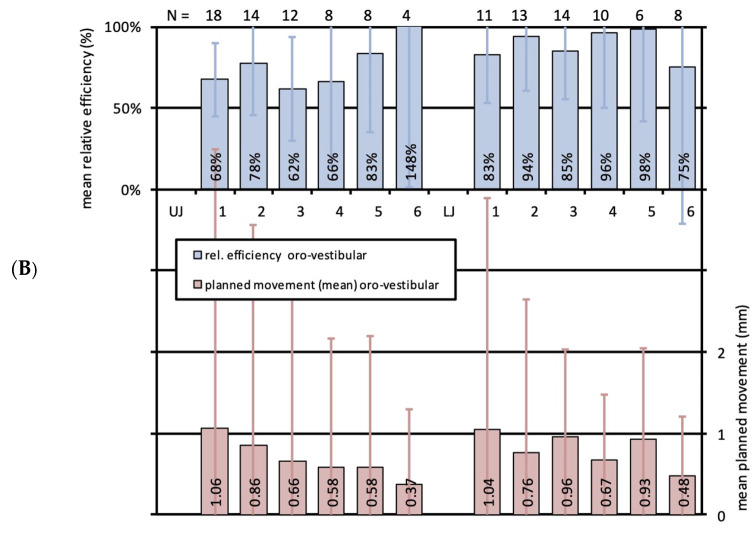
(**A**) Bar graph illustrating the relative effectivity of translational orovestibular tooth movements for Group 1 (adults, Invisalign^®^). Upper bars (blue) indicate the relative effectivity and the error in percent for each tooth of the upper (UJ, left) and the lower (LJ, right) jaw. Lower bars (red) show the mean planned movements in mm with standard deviations (SD). N-numbers of the corresponding mean values are specified on top of the illustration; (**B**) Bar graph illustrating the relative effectivity of translational orovestibular tooth movements for Group 2 (adolescents, Invisalign^®^ Teen). Upper bars (blue) indicate the relative effectivity and the error in percent for each tooth of the upper (UJ, left) and the lower (LJ, right) jaw. Lower bars (red) show the mean planned movements in mm with standard deviations (SD). N-numbers of the corresponding mean values are specified on top of the illustration.

As seen for orovestibular movements of the whole cohort, both adult patients of Group 1 and adolescents of Group 2 displayed the same pattern of planned movement amounts for the different tooth groups when regarded separately. Interestingly, Group 2 manifested a moderate effectivity for upper first and second incisors (68%, 78%), whereas Group 1 presented a high effectivity of 90% and 87% for those teeth. Performance was reciprocal for upper canines, as they remained corrected below the expected result for Group 1 (34%), but at least featured a moderate effectivity for Group 2 (62%). For the rest of the teeth, values were approximately congruent for both groups, showing slightly better values for most of the lower teeth for Group 2. Results are displayed in Figure 5A,B. No significant differences between groups could be noted.

### 3.4. Effectiveness of Orovestibular Tipping in Upper and Lower Jaws for Adolescent and Adult Patients

**Figure 6:**
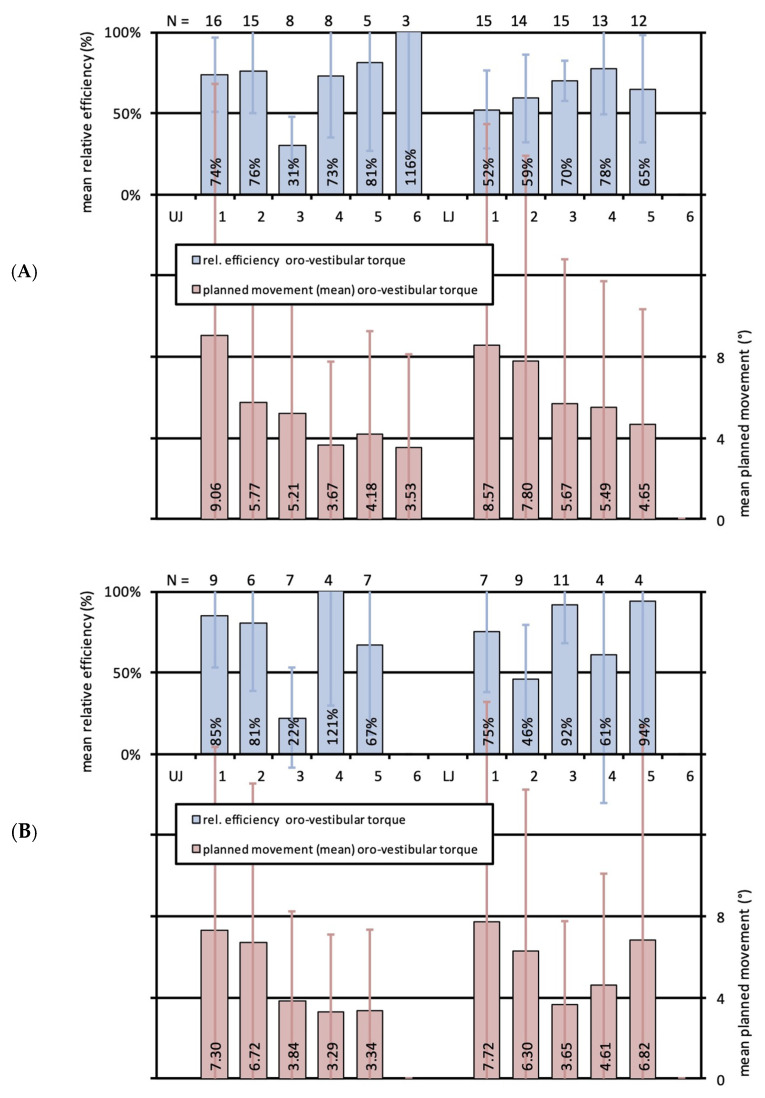
(**A**) Bar graph illustrating the relative effectivity of rotations around the y-axis, equivalent to orovestibular torque, for Group 1 (adults, Invisalign^®^). Upper bars (blue) indicate the relative effectivity and the error in percent for each tooth of the upper (UJ, left) and the lower (LJ, right) jaw. Lower bars (red) show the mean planned movements in mm with standard deviations (SD). N-numbers of the corresponding mean values are specified on top of the illustration; (**B**) Bar graph illustrating the relative effectivity of rotations around the y-axis, equivalent to orovestibular torque, for Group 2 (adolescents, Invisalign^®^ Teen). Upper bars (blue) indicate the relative effectivity and the error in percent for each tooth of the upper (UJ, left) and the lower (LJ, right) jaw. Lower bars (red) show the mean planned movements in mm with standard deviations (SD). N-numbers of the corresponding mean values are specified on top of the illustration.

In concordance with the results for the whole cohort, crown tipping was most pronounced for the first upper and lower incisors in both groups. Interestingly, performance was much better in Group 2 for upper and lower first incisors, upper second incisors and upper second premolars. Lower second premolars and lower canines even featured a very high effectivity of 94% and 92%, which is reflected in Figure 6B. Lower molars in Group 1 and upper plus lower molars in Group 2 could not be incorporated into analyses due to a sample size below three. In Group 2, a correction below the expected result was seen for upper canines (22%) and lower second incisors (46%), and a moderate effectivity for lower first (61%) and upper second premolars (67%) with correction values of 4.6° and 3.3°, respectively. For Group 1, the very high effectivity of 116% was striking for the upper molars, with a mean planned orovestibular tipping movement of 3.5°, which is shown in Figure 6A. However, the amount of data is moderate with a sample size of *n* = 3. Values were better for the upper incisors and second premolars compared to the lower ones, and vice versa for the lower canines and first premolars. Upper canines could only provide a correction below the expected result of 31% for a movement of 5.2°, exhibiting a small standard deviation. Analyses evidenced that statistical outcomes did not significantly differ between groups.

## 4. Discussion

Against the background of its effectivity, the spectrum of aligner orthodontic treatment indication represents a permanent and controversial discussion in the literature. Particularly with regard to the realization of arch expanding and compressing tooth movements in orovestibular direction, the data availability is insufficient. The present study aimed at surveying the conformity of planned and actually achieved orovestibular tooth movements individually for each tooth from the first incisor up to the first molar in both jaws. Consequently, this work is, to our knowledge, the first one analyzing this question comprehensively and in this complexity for the whole dentition. Our investigations revealed that the efficiency of orovestibular movements was excellent, with no statistical significance in relation to jaw, tooth type or Invisalign^®^ system. Translational tooth movements in the mandible were highly effective and outstanding for premolars (91–98%). Translational tooth movements in the upper jaw were successful for incisors and premolars, but less effective for canines and molars. Almost all teeth were moderately or very effectively corrected by crown tipping, with better results for mandibular teeth (70–92%) than maxillary teeth (22–31%), as well as upper anterior teeth (81–85%) and lower canines (92%) in adolescents. 

The underlying assumptions of our investigations were a varying performance of aligners in effecting arch expansion and compression movements, which is caused on the one hand by the different bone structure of maxilla and mandible, and on the other hand by the varying root anatomy of tooth groups. Furthermore, we gave attention to potential differences in the treatment effectivity between the conventional Invisalign^®^ system and Invisalign^®^ Teen, which has never been analyzed so far. Our investigations revealed that orovestibular tooth movements effecting arch expansion or compression can be differentially realized via translational movement or via crown tipping. Key parameters are the tooth specimen as much as patient age, namely teenager or adult. However, the overall performance of aligners regarding the tooth movements investigated was very good and differences were not statistically significant for the tooth types of both jaws, which confirms the aligner treatment as a very valid treatment option. Thus, our null hypothesis that there are no statistically significant differences between treatment outcomes and jaw, tooth group or aligner system could not be refuted by the results of the study.

Translations in oral and vestibular direction could be realized with high or even very high effectivity for the lower jaw of adolescents and likewise for adults, except the lower molars of the latter ones. Particularly, the performance for translational movements was outstanding for first and second lower premolars (91–98%). In the upper jaw, these translational movements could be successfully conducted in the front and premolar region as well, but are less effective for the canines and molars. These results are in concordance with the observations of Kravitz et al. [26], postulating that translational movements in orovestibular direction are best convertible with aligners. However, this study exclusively focused on the front region and additionally only reached values of 41% effectivity in the mean. At this point, it should be recalled that some authors suppose a pretense of bodily translational movement by an actual tipping [16,17]. As the focus of our study exclusively involved crown movements, this thesis can neither be supported nor neglected.

When regarding the implementation of orovestibular crown tipping in our study, almost all teeth could be moderately or very effectively corrected. Lower jaw performance was much better in the feasibility of canine movements (70–92%) compared to upper jaws (22–31%). Generally, tipping movement implementation was more effective in adolescents than in adults for selected teeth, namely upper front teeth and lower canines. Former investigations found similar results and concluded a good feasibility of protrusions and accordingly tipping in orovestibular direction, though this work also exclusively regarding the front region and not the whole dentition, as done in our study [6]. Contrarily, other investigations only found effectivity between 40–47% or reasoned an inferior performance of aligners compared to multibracket appliances for these movements [10,26]. 

When discussing the present data, some limitations have to be kept in mind. The superimposition works best with identical geometries, but during data processing by Align Technology Inc., the tooth surfaces are smoothed and soft tissues are replaced by an idealized geometry. The clinical data on the other hand represent raw scan data without smoothing. This accounts for both methods of clinical recording of the actual situation, namely scanning of plaster casts and direct intraoral scanning. These differences might lead to an additional measurement noise in the determination of tooth movements. However, there is no way to avoid this methodological pitfall, as the ClinCheck^®^ models provide the exclusive method to determine the planned tooth movements. We also aimed at achieving perfect superimposition conditions. Therefore, we exclusively included cases with at least three unmoved teeth per jaw, and additionally excluded those which regardless exhibited movement on these presumably stable teeth. 

Secondly, a methodological drawback of the study is the fact that the principle of superimposition is error-prone regarding the interpretation of results. However, the chosen type of examination is the only methodology available to date for addressing questions such as those in this study. Thus, the principle of superimposition represents the most appropriate research design applicable for the analyses to perform. The susceptibility to errors is evidenced by some of our measurements featuring orovestibular efficiency ratios exceeding 100%, which is impossible and a consequence of inaccuracies in the superimposition technique. However, as stated above, these system-immanent errors cannot be prevented, but can only be consciously perceived as false values and excluded from the interpretation of the data. 

As another point, it certainly has to be considered that our patients were treated with Invisalign^®^ Exceed30 (EX30) aligner material preceding SmartTrack (LD30), which represents the next generation of aligner material [28]. As LD30 exhibits higher elasticity, better arch adaptability, greater consistency of orthodontic force application and lower warping after use than EX30, current aligners presumably perform even better in efficiency of orovestibular movements [28]. Nonetheless, our study is the first and only one that evaluated orovestibular movements to this extent, applying superimposition techniques and investigating the whole jaws with each single tooth.

We found that lower canines and premolars have more orovestibular relative efficiency than lower incisors, despite the fact that incisors have smaller roots and can therefore be moved more easily. Here, the tooth geometry will not have a decisive influence on the efficiency of the movement, as premolars in particular, but also canines, have a better grip than anterior teeth, but this is compensated for when planning the tooth movement by attachments that change the tooth geometry to allow a better force transmission. This observed phenomenon could be due to a methodological drawback of the study, which is the origin used to evaluate the rotations. As the shape of the roots cannot be determined in this study due to the technical limitations dictated by the ClinCheck^®^, we cannot use the center of resistance to describe tooth movements, especially the rotations. To overcome this burden, we instead used a point defined by the highest central point of each crown extracted from the initial model M.in to calculate the rotations. This can be error-prone for analytical outcomes when the tooth is tilted, as the center of the tooth crown will also move and can be misleadingly interpreted as an orovestibular displacement, even though it is a tilt of the tooth. This can influence the relationship between physical translations and compensatory rotations, but as we used the same origin for both calculations, namely planned and clinical, this systemically necessary procedure is acceptable. Nevertheless, with the future gain of further technical possibilities, such system-related limitations will be overcome in favor of higher accuracy in data calculations reflecting clinical practice even more precisely.

A recent study also investigated arch expansion with aligners and their effect on different stride and torque and found that the posterior teeth cause a certain buccal tilt when the maxillary arch is expanded [29]. Compliant with our investigations, this study also examined the effects on all tooth types, but only in the upper jaw, whereas data on the lower jaw are not available. Furthermore, the examinations were carried out using finite element analysis, which does not provide real clinical data like ours. This also accounts for a current study likewise dealing with expansion efficiency and propagating that torque compensation should be implemented in the planning to enhance torque control during arch expansion, which also applied finite element analysis and only objected the upper jaw [30]. Another very recent study evaluated aligner performance regarding the efficiency and predictability of expansion, here in a retrospective study like ours likewise comparing maxillary and mandibular pre-treatment, planned treatment and post-treatment models [31]. They found that the greatest expansion was found in both the upper and lower premolars, and that aligners are effective for simultaneous intraoral expansion in both jaws. The weakness of this study is that linear measurements of the interdental widths were recorded for the examinations, including the intercanine width between the cusp tips, the interpremolar widths between the palatal cusp tips of the first and second premolars and the intermolar width between the tips of the mesopalatine cusps of the first molars. However, this method is far less accurate and much more error-prone than our analysis of individual tooth movements, which involved superimposing individual scatter plots. Another retrospective study and systematic review focused on the efficiency of Invisalign First^®^ regarding the quality of expansion movements in the mixed dentition [32]. However, the digital models from pre-treatment, ClinCheck^®^ -predicted tooth positions and post-treatment were again only analyzed for the maxillary dental arch width and expansion efficiency. Furthermore, measurements were again linear with reference points on the mesiopalatal cusp tip of the temporary and permanent molars, palatal cusp tip of the premolars and cusp tip of temporary and permanent canine, lacking the necessary accuracy as described above.

In summary, this retrospective study combines the topic of aligner efficiency of orovestibular movements and systematically implements it for the entire dentition by applying the 3D superimposition methodology. The results obtained with this advanced and seminal superimposition technique have the potential for clinical contribution to improved and contemporary orthodontic treatment planning, optimized aligner performance and, thus, successful treatment outcomes and patient satisfaction. Directions for future research in this field that have emerged from our study are certainly the even more targeted use of specific auxiliary means such as attachments according to individual patient needs in order to make tooth movement even more efficient, as well as continuous optimization of the already more than sufficient, but still perfectible, material properties of the aligners.

## 5. Conclusions

Taken together, translational orovestibular movements can be very effectively realized, and additional support in arch expansion or compression therapies can be successfully provided by crown tipping, which accounts for all teeth in both jaws, regardless of the Invisalign^®^ system applied. Thus, our hypothesis that these aspects might negatively impact the efficiency and treatment success of orovestibular tooth movement can be neglected.

Aligners secure effective translational orovestibular movements. Nonetheless, treatment planning should take individual parameters into account.

These data provide the basis and represent a benchmark for future investigations in this field of research. Furthermore, the results of our study serve as reliable basis for treatment planning by practicing clinicians that can be implemented in patient therapy with aligners.

## Data Availability

The raw data supporting the conclusions of this article will be made available by the authors on request.
